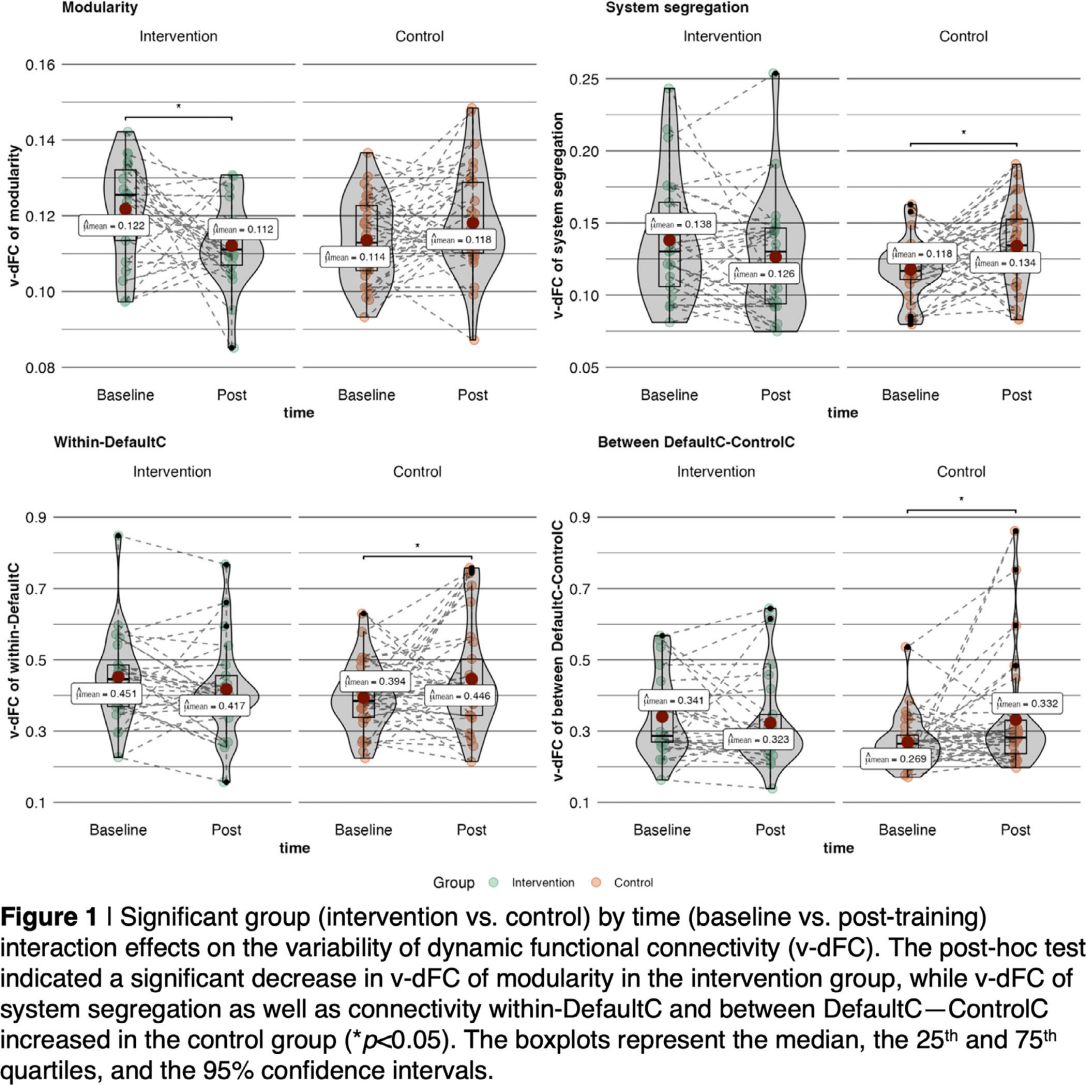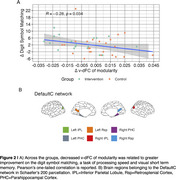# Intervention‐related changes in variability of dynamic functional connectivity and the relationship to cognition in older adults at risk for Alzheimer's disease

**DOI:** 10.1002/alz70856_105792

**Published:** 2026-01-10

**Authors:** Paulina Skolasinska, Caitlin S. Walker, Adrian Noriega de la Colina, Alfie Wearn, Colleen S. Hughes, Roni Setton, Jillian Caplan, Laurence Côté, Kayla Williams, Sofia Ricciardelli, Linda Li, Carolynn Boulanger, Nagashree Thovinakere, Garance Barnoin, Sarah Elbaz, Mitchell Bennett, Shania Fock Ka Bao, Ryan Kara, Nicolas Lavoie, Maggie Nguyen, Franciska Otaner, Helen Pallett‐Wiesel, Johanie Victoria Piché, Andreanne Powers, Christine Dery, Prantik Kundu, Ilana R Leppert, Jennifer Tremblay‐Mercier, Judes Poirier, Sylvia Villeneuve, Arthur F. Kramer, R. Nathan Spreng, Maiya R. Geddes, PREVENT‐AD Research Group

**Affiliations:** ^1^ Montreal Neurological Institute‐Hospital (The Neuro), McGill University, Montreal, QC, Canada; ^2^ Centre for Studies on Prevention of Alzheimer's disease (StoP‐AD Centre), Montreal, QC, Canada; ^3^ Douglas Mental Health University Institute, Montreal, QC, Canada; ^4^ Massachusetts Institute of Technology, Cambridge, MA, USA; ^5^ Montreal Neurological Institute, McGill University, Montreal, QC, Canada; ^6^ Indiana University, Bloomington, IN, USA; ^7^ Harvard University, Cambridge, MA, USA; ^8^ Faculty of Medicine and Health Sciences, McGill University, Montreal, QC, Canada; ^9^ The Neuro, Faculty of Medicine, McGill University, Montreal, QC, Canada; ^10^ Department of Pharmacology and Therapeutics, McGill University, Montreal, QC, Canada; ^11^ Rotman Research Institute, University of Toronto, Toronto, ON, Canada; ^12^ McGill University, Montreal, QC, Canada; ^13^ Faculty of Medicine, Laval University, Quebec City, QC, Canada; ^14^ Faculty of Pharmacy, Université de Montréal, Montreal, QC, Canada; ^15^ School of Physical and Occupational Therapy, Faculty of Medicine and Health Sciences, McGill University, Montreal, QC, Canada; ^16^ Centre for Studies in the Prevention of Alzheimer's Disease, Douglas Mental Health Institute, McGill University, Montreal, QC, Canada; ^17^ BioMedical Engineering and Imaging Institute, Icahn School of Medicine at Mount Sinai, New York, NY, USA; ^18^ Friedman Brain Institute, Icahn School of Medicine at Mount Sinai, New York, NY, USA; ^19^ McConnell Brain Imaging Center, McGill University, Montreal, QC, Canada; ^20^ Centre for Studies on Prevention of Alzheimer's Disease (StoP‐AD Centre), Montreal, QC, Canada; ^21^ McConnell Brain Imaging Centre (BIC), MNI, Faculty of Medicine, McGill University, Montreal, QC, Canada; ^22^ StoP‐AD Centre, Douglas Mental Health Institute Research Centre, Montreal, QC, Canada; ^23^ Beckman Institute, University of Illinois, Urbana, IL, USA; ^24^ Center for Cognitive & Brain Health, Northeastern University, Boston, MA, USA; ^25^ McGill University Research Centre for Studies in Aging, McGill University, Montreal, QC, Canada

## Abstract

**Background:**

Aging is associated with a decline in specific cognitive abilities and increased variability of dynamic functional connectivity (v‐dFC; Jauny et al., 2022), across the whole brain (Yang et al., 2023) and in the default network (Douw et al., 2016; Madhyastha & Grabowski, 2014). We investigated the extent to which age‐related changes in v‐dFC would be mitigated by an intervention to enhance physical activity in older adults at risk for Alzheimer's disease (AD) and whether the changes in v‐dFC were related to cognitive improvements.

**Method:**

50 participants from the PREVENT‐AD longitudinal cohort (Tremblay‐Mercier et al., 2021) with a family history of AD were assigned to the intervention (*N* = 21; M_age_=69.8 years) or the active control group (*N* = 29; M_age_=70.3 years) for a four‐week randomized controlled trial. Multi‐echo gradient‐echo EPI sequence was used to acquire resting‐state functional MRI data at baseline and post‐intervention. Using Schaefer's 200 parcellation across Yeo's 17 networks, the dFC matrices were constructed with the Multiplication of Temporal Derivatives method (Shine et al., 2015; 10 TR overlapping windows). Modularity, system segregation (Chan et al., 2014), within‐network and between‐network FC of the DefaultA/B/C and ControlA/B/C networks were calculated for each dFC matrix. V‐dFC was defined as the standard deviation of these measures. Group by time interactions effects on v‐dFC were estimated after controlling for age, education, sex, motion and *APOE4* carriership status. Change scores in v‐dFC measures were correlated with changes in cognitive performance on digit span and digit symbol matching tasks.

**Result:**

The intervention group showed decreased modularity and maintained system segregation within‐DefaultC, between DefaultC—ControlC v‐dFC, compared to the control group who showed increased v‐dFC (Figure 1) after the intervention period relative to baseline. The baseline to post‐intervention decrease in v‐dFC of modularity was related to concurrent improvement in digit symbol matching task performance (Figure 2).

**Conclusion:**

An intervention to enhance physical activity mitigated age‐related increases in v‐dFC network segregation and connectivity of the DefaultC subnetwork. DefaultC corresponds to the medial temporal lobe subsystem (Andrews‐Hanna et al., 2010), including the parahippocampal, retrosplenial and inferior parietal regions, which are highly vulnerable to AD pathology (Buckner et al., 2005).